# Understanding the complexity of *Tityus serrulatus* venom: A focus on high molecular weight components

**DOI:** 10.1590/1678-9199-JVATITD-2023-0046

**Published:** 2024-01-22

**Authors:** Isadora Sousa de Oliveira, Nicoly Malachize Alano-da-Silva, Isabela Gobbo Ferreira, Felipe Augusto Cerni, Jacqueline de Almeida Gonçalves Sachett, Wuelton Marcelo Monteiro, Manuela Berto Pucca, Eliane Candiani Arantes

**Affiliations:** 1 Department of BioMolecular Sciences, School of Pharmaceutical Sciences of Ribeirão Preto, University of São Paulo, Ribeirão Preto, SP, Brazil.; 2 Department of Biotechnology and Biomedicine, Technical University of Denmark, Kongens Lyngby, Denmark.; 3 Health and Sciences Postgraduate Program, Federal University of Roraima, Boa Vista, RR, Brazil.; 4 School of Health Sciences, Amazonas State University, Manaus, AM, Brazil.; 5 Department of Teaching and Research, Dr. Heitor Vieira Dourado Tropical Medicine Foundation, Manaus, AM, Brazil.; 6 Department of Clinical Analysis, School of Pharmaceutical Sciences, São Paulo State University, Araraquara, SP, Brazil.

**Keywords:** Tityus serrulatus, Proteases, Hyaluronidase, Phospholipase, Cysteine-rich secretory proteins

## Abstract

*Tityus serrulatus* scorpion is responsible for a significant number of envenomings in Brazil, ranging from mild to severe, and in some cases, leading to fatalities. While supportive care is the primary treatment modality, moderate and severe cases require antivenom administration despite potential limitations and adverse effects. The remarkable proliferation of *T. serrulatus* scorpions, attributed to their biology and asexual reproduction, contributes to a high incidence of envenomation. *T. serrulatus* scorpion venom predominantly consists of short proteins acting as neurotoxins (α and β), that primarily target ion channels. Nevertheless, high molecular weight compounds, including metalloproteases, serine proteases, phospholipases, and hyaluronidases, are also present in the venom. These compounds play a crucial role in envenomation, influencing the severity of symptoms and the spread of venom. This review endeavors to comprehensively understand the *T. serrulatus* scorpion venom by elucidating the primary high molecular weight compounds and exploring their potential contributions to envenomation. Understanding these compounds' mechanisms of action can aid in developing more effective treatments and prevention strategies, ultimately mitigating the impact of scorpion envenomation on public health in Brazil.

## Background

**Figure 1.**
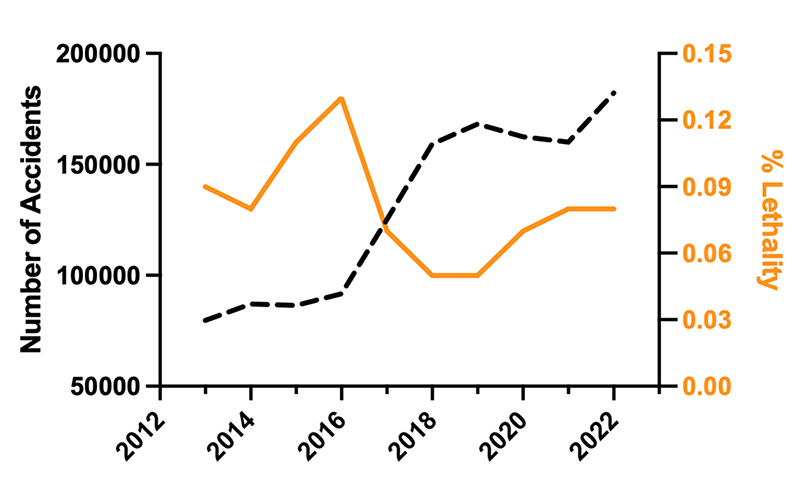
Trends in scorpionism in Brazil over the past decade. The left y-axis represents the number of scorpion sting incidents, while the right y-axis represents the lethality rate (%), calculated by the equation $\mathrm{Lethality rate (\%)}=\frac{\mathrm{number of deaths}}{\mathrm{number of cases}}x100$ . The years 2020, 2021, and 2022 are still subject to review [2].

Scorpion stings represent a major public health challenge across the globe, with Brazil being one of the most severely impacted countries [1]. Despite the relatively low lethality rate of scorpionism in Brazil, the number of incidents in the country has risen dramatically over the past decade. Indeed, the number of scorpion sting incidents has increased by over 200% in the last ten years (Figure 1), from approximately 80,000 incidents in 2013 to more than 180,000 in 2022 [2]. The prevalence of scorpions in urban areas, coupled with factors such as deforestation and urbanization, has led to a surge in human-scorpion interactions. These interactions, often resulting in stings, have raised significant public health concerns across the country. The rise in scorpionism has prompted local authorities and healthcare providers to bolster their efforts in terms of prevention, treatment, and education to better address this growing issue and ensure the safety and well-being of the Brazilian population [3,4]. This trend is a cause for concern and underscores the need for effective prevention and treatment strategies to address this growing public health issue. Among the various scorpion species found in Brazil, those belonging to the *Tityus* genus are of medical importance. The *Tityus serrulatus* scorpion is responsible for the most severe cases of envenomation and fatalities [5,6], especially in areas of human population densities [7]. This scorpion's venom is a complex mixture of various molecules, including low molecular weight peptides such as neurotoxins and high molecular weight proteins such as enzymes [6,8]. While several studies have explored the toxic and mechanistic effects of neurotoxins, there is a lack of understanding regarding the role of high molecular weight proteins in the pathogenesis of *T. serrulatus* envenoming.

This review is dedicated to offering a comprehensive insight into the T. serrulatus scorpion, shedding light on the intricate array of molecules present in its venom. Our primary focus lies on the high molecular weight proteins, recognized for their significant involvement in the pathogenesis of envenomation. It is crucial to clarify that, in this context, we define high molecular weight proteins as those exceeding 14 kDa, constituting approximately 20-25% of the venom composition [9]. Furthermore, this review will delve into the prominent high molecular weight proteins within T. serrulatus venom, underscoring the imperative need for further research to fully harness their potential applications.

## Tityus serrulatus envenomation and treatment

T. serrulatus sting can lead to a wide range of clinical manifestations, varying from mild to severe, and can even result in death in some cases. Local symptoms such as pain, edema, erythema, sudoresis, and paresthesia are among the most commonly reported. These symptoms usually appear within hours of the sting and can last for several days. In addition to local symptoms, systemic manifestations can also occur. Tachycardia, diaphoresis, profuse sweating, psychomotor agitation, tremors, nausea, vomiting, sialorrhea, arterial hypertension, or hypotension are some systemic symptoms observed after T. serrulatus envenoming [3,5,6]. In severe systemic manifestations, other clinical manifestations may occur, including acute pulmonary edema, cardiovascular collapse, cardiac arrhythmia, congestive heart failure, and shock. In addition to clinical evaluation, complementary imaging, and biochemical tests are important for monitoring cases through an electrocardiogram, chest X-ray, echocardiogram, and biochemical tests to assess elevated creatine phosphokinase (CPK), and its MB fraction, hyperglycemia, hyperamylasemia, hypokalemia, and hyponatremia [10,11]. These symptoms have the potential to be life-threatening and necessitate prompt medical attention. Generally, the severity of T. serrulatus envenoming depends on the amount of venom injected, the time between the sting and medical intervention, and the individual's age and health status. Indeed, children under six and, less frequently, the elderly with comorbidities are more seriously affected and are related to most deaths [2,12].

A study was conducted on children under the age of 15 who experienced severe symptoms after being stung by T. serrulatus and were subsequently admitted to the intensive care unit. The study found that the most common symptoms reported by these children were tachycardia, sweating, and agitation. Furthermore, abnormal liver function tests were observed, with significant increases in aspartate aminotransferase (AST) and alanine aminotransferase (ALT) levels. There was also a high incidence of pulmonary edema, which in rare cases progressed to respiratory failure and even death. These findings underscore the importance of promptly recognizing and aggressively managing severe T. serrulatus envenomation in children, especially those with abnormal liver function tests and pulmonary edema [13].

The T. serrulatus envenoming treatment is primarily supportive, and early administration of analgesics, antihistamines, and benzodiazepines can help alleviate symptoms and prevent complications [14]. In mild cases, characterized only by local signs and symptoms, antivenom usage is not recommended, only in symptomatic treatment, and observation of the clinical condition for at least 6 hours after the incident is advised [15]. In moderate and severe cases, Brazil has two different antivenoms available: the arachnid antivenom (each vial with 5 mL contains a fraction of heterologous F(ab’)_2_ immunoglobulins that neutralize a minimum of 15.0 minimum lethal dose (MLD) of Loxosceles gaucho venom, 1.5 MLD of Phoneutria nigriventer venom, and 1.5 MLD of T. serrulatus venom per mL [16]) and the scorpion antivenom (each vial with 5 mL contains a fraction of heterologous F(ab’)_2_ immunoglobulins that neutralize a minimum of 5.0 mg of T. serrulatus reference venom [17]). For moderate cases, patients with signs of intense local pain associated with some manifestations are considered, thus, two to three vials of antivenom are administered. In severe cases, with the presence of more intense and severe local and systemic signs related to the respiratory and cardiovascular systems, four to six vials of antivenom are recommended [15]. 

Additional treatments may also be necessary, such as vasodilators, anti-arrhythmic agents, and inotropes. Therefore, healthcare professionals must thoroughly understand the clinical presentation and management of T. serrulatus envenoming to ensure optimal patient outcomes [6]. It is crucial to note that the use of antivenom should be based on clinical criteria, such as the severity of envenomation, rather than solely on the confirmation of a scorpion sting. Although antivenom is considered the mainstay of treatment for moderate and severe T. serrulatus envenoming, it is essential to understand that the use of heterologous antivenom has some limitations and can lead to adverse effects [18]. As such, a careful risk-benefit assessment should be made before administering antivenom.

## Tityus serrulatus biology

**Figure 2.**
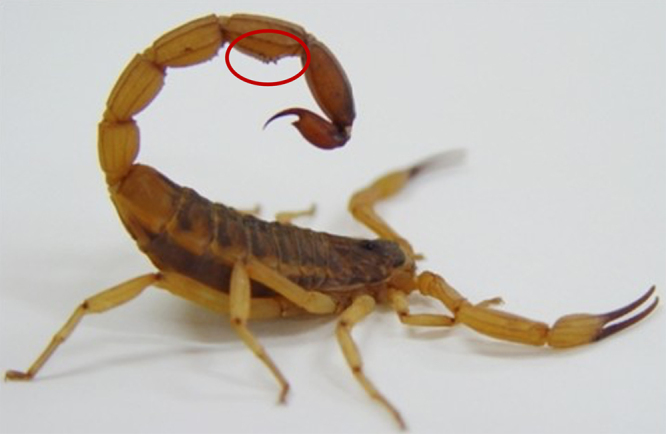
Tityus serrulatus scorpion**.** Tityus serrulatus, commonly measuring between 7-9 centimeters (approximately 2.75-3.5 inches) in length, is characterized by its brown to dark brown color. The species name "serrulatus" is derived from the Portuguese term "serrilha", which refers to the serrated feature in its tail, indicated by a red circle in the image, setting it apart as a distinctive anatomical hallmark [21].

Popularly known as the yellow scorpion, T. serrulatus epitomizes a highly specialized species adapted to tropical and subtropical Brazilian ecosystems. Belonging to the arachnid class within the subphylum Chelicerata, scorpions possess four pairs of appendages distributed along their segmented body, comprising the prosoma (cephalothorax) and the opisthosoma (abdomen and tail). T. serrulatus exhibits well-developed chelicerae and pedipalps in the anterior cephalothorax, pivotal in facilitating the feeding process. In the terminal section of the opisthosoma, referred to as the telson, the venom-secreting glands are housing the stinger, a specialized apparatus responsible for venom delivery. Additionally, T. serrulatus showcases a distinctive anatomical feature in the tail (Figure 2), characterized by diminutive tooth-like structures or serrations, which have warranted the species' designation of “serrulatus” [6,19,20].

Parthenogenesis, a form of asexual reproduction, emerges as a pivotal factor propelling the proliferation of T. serrulatus. Within this process, eggs undergo development without the need for fertilization, a relatively uncommon phenomenon in nature, albeit observed in select scorpion species. Despite reports of male T. serrulatus individuals, the extent of sexual reproduction in this species remains incompletely elucidated, as the preponderance of females strongly suggests a propensity towards parthenogenetic reproduction as the primary reproductive mode [20,22].

Similar to other scorpion species, T. serrulatus showcases remarkable resilience during prolonged periods of food deprivation, with reports documenting individuals surviving up to 400 days without sustenance. Nevertheless, this endurance does not extend to the absence of water access, which emerges as a critical determinant for the species' sustenance and survival [23].

Consequently, the synergistic combination of asexual reproduction and resistance to starvation contributes to the rapid expansion of T. serrulatus populations, thereby extending their habitat range and heightening the potential for human encounters and associated incidents [20].

## Neurotoxicity triggered by low molecular weight compounds

T. serrulatus venom is a highly intricate combination of various compounds. It serves as a valuable repository of small neurotoxic proteins (refer to Table 1), playing crucial roles in prey capture, defense against predators [24,25], and interacting with diverse ionic channels in excitable membranes, contributing to their biological effects [26].

**Table 1.** Small neurotoxins found on Tityus serrulatus scorpion venom that target ion channels.

Toxin	Target	Mechanism of action	Ref.
Ts1	Nav	Shifts the voltage of activation toward more negative potentials	[27]
Ts2	Nav	Inhibit the inactivation of the activated channels, blocking neuronal transmission; induces macrophage activation and production of immune mediators	[27,28]
Ts3	Nav	Inhibit inactivation of the activated channels, blocking neuronal transmission	[29]
Ts4	Nav	Induces release of neurotransmitters glutamic acid and gamma-aminobutyric acid; induces allergic reaction	[30]
Ts5	Nav and Kv	Inhibiting inactivation and blocking neuronal transmission; increases potassium permeability	[31]
Ts6	Kv	Block channels; induces macrophage activation and production of immune mediators	[28,32]
Ts7	Kv	Blocks multiple voltage-gated potassium channel subtypes; blocks Kv1.3 channel by occluding the pore	[32]
Ts8	Kv	Blocks potassium channels; inhibits Kv4.2 channel and produces nociception in vivo	[33]
Ts9	Kv	Blocks small-conductance calcium-activated potassium channels	[34]
Ts11	Kv	Blocks potassium channels at different proportions	[35]
Ts12	Kv	Blocks potassium channels	[35]
Ts15	Kv	Preferentially blocks Kv1.2, Kv1.3 and Kv2.1	[36,37]
Ts17	Nav	Change the kinetics of Nav1.2 and Nav1.5	[38]
Ts19 frag-II	Kv	Block Kv1.2	[39]
Ts32	Cav	Increases intracellular Ca^2+^ release	[40]

Voltage-gated Na^+^ channel toxins are the primary and highly reactive components responsible for the toxic effects of scorpion envenoming. These toxins are long-chain peptides and can be categorized into two classes: α- and β-scorpion neurotoxins [41,42]. The α-toxins specifically bind to site three, located on extracellular loops S3-S4 of domain IV of the ion channel. This binding hinders or even blocks the inactivation mechanism of these channels, resulting in their prolonged activation [43]. On the other hand, β-toxins bind to site four of the channel, immobilizing it and keeping it in the activated position [44,45]. The α and β-toxins, such as Ts1-5, Ts17, Ts18, Ts26-28, and Ts30, have a specific affinity for Na^+^ channels, thereby modulating the activated channels. Some of these toxins, like Ts5, may also interfere with the permeability of K^+^ channels [46]. 

K^+^ channel neurotoxins, including Ts6-9, Ts11, Ts12, Ts15, Ts16, and Ts19-25, exhibit inhibitory or blocking effects on K^+^ channels [26,47]. Specifically, Ts11-13, which were described by Pimenta et al. [48], are 29 amino-acid peptide sequences that contain four disulfide bridges. Another noteworthy toxin is Ts32, as reported by De Oliveira et al. [49]. Ts32 is a cell-penetrating peptide and represents the only Ca^2+^-specific toxin identified in T. serrulatus venom thus far. This particular toxin is capable of increasing intracellular Ca^2+^ release and holds promising biotechnological potential for the treatment of cancer cells [40,49]. 

Verano-Braga et al. [50] employed a proteomic approach to identify a novel group of peptides within T. serrulatus venom, referred to as hypotensins. These peptides are characterized by their random-coiled linear structure and possess a similar amino acid signature to bradykinin-potentiating peptides. The study revealed that hypotensins exhibit hypotensive effects and induce endothelium-dependent vasorelaxation, which is mediated by the release of nitric oxide (NO) [50].

Short-chain toxins found in T. serrulatus venom consist of 30-32 amino acid residues, primarily held together by three disulfide bridges, and this family of peptides constitutes a significant group that primarily targets K^+^ channels [26]. These toxins exhibit diverse biological activities, including but not limited to bradykinin-potentiating effects, antimicrobial properties, hemolytic activity, hypotensive effects (hypotensins), immune-modulating capabilities, and hormone-like activities [51]. Their wide range of biological activities highlights their versatility and potential for various applications.

Regarding the omic analysis of T. serrulatus venom, there have been two notable reports involving transcriptomic and proteomic analyses. The more recent study, conducted by De Oliveira et al. [49], identified new peptides capable of modulating ion channels. This analysis shed light on previously unknown components of the venom.

Additionally, T. serrulatus venom has been found to contain various low molecular weight components, which include antimicrobial peptides, hypotensins (previously mentioned), C-type natriuretic peptides, and non-disulfide peptides with angiotensin-converting enzyme inhibitor activity [52]. These findings demonstrate the diverse range of bioactive molecules present in T. serrulatus venom and their potential for various therapeutic applications.

According to the transcriptomic analysis conducted by Kalapothakis et al. [52], the most abundant toxin types in T. serrulatus venom are Na^+^ and K^+^ channel toxins, accounting for 45.24% and 38.10% of the total toxins, respectively. In addition, nine novel putative toxin sequences (Ts33-Ts41) were identified, being that, Ts33-35, Ts37, and Ts38 possess the conserved Toxin_3 domain, suggesting their potential action on Na^+^ channels. Ts36, Ts39, and Ts40 do not exhibit this domain, but show similarities, respectively, to toxins such as JAW07013.1 from T. serrulatus, AGT39262.1 from Mesobuthus eupeus, and ADY39581.1 from Hottentotta judaicus, respectively [52].

## High molecular weight compounds and how they could interfere in the envenoming

**Figure 3:**
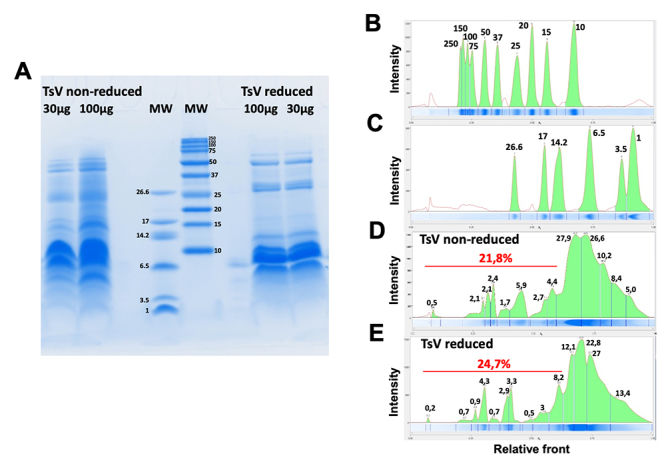
Tricine-SDS-PAGE and densitometry of Tityus serrulatus scorpion venom (TsV). **(A)** Tricine-SDS-PAGE profiles. Lanes 1 and 2: non-reduced TsV, 30 and 100 µg, respectively; Lanes 3 and 4: molecular weight markers (MW); Lanes 5 and 6: reduced TsV, 30 and 100 µg, respectively. **(B-E)** Densitometry of the bands obtained from Tricine-SDS-PAGE. **(B-C)** Densitometry showing the molecular weight of the standards. **(D-E)** Densitometry showing the percentage of the non-reduced and reduced TsV bands, respectively. Tricine-SDS-PAGE was performed as described by Schägger and von Jagow [53]. The densitometry analysis was performed using a densitometer Image Lab™ Software.

Several studies have been conducted to investigate the venom of T. serrulatus, using transcriptomes and proteomics techniques. These studies have significantly contributed to our understanding of the venom's composition. Most proteins present in the T. serrulatus venom are neurotoxins with action on ion channels and molecular weights lower than 14 kDa. However, the venom also has many enzymes and other components with molecular weights higher than 14 kDa (~20-25%, Figure 3 A-E ), still little characterized. Therefore, this work highlights the main venom compounds with molecular weight higher than 14 kDa.

Notably, Alvarenga et al. [54] identified various high molecular weight components using transcriptomic analysis, including antarease, zinc metalloproteases, proteins rich in cysteine, hyaluronidase, and phospholipase A_2_ (PLA_2_). Similarly, De Oliveira et al. [49] identified metalloproteinase, hyaluronidase, cysteine-rich secretory protein (CRISP), PLA_2_, phospholipase C (PLC), and phospholipase D (PLD) through their research. Additionally, Amorim et al. [8] studies revealed the presence of metalloproteinases, CRISPs, phospholipases, and phosphodiesterases (PDE). 

Recently, Kalapothakis et al. [52] reported a novel transcriptomic approach that unveiled the presence of new components in the venom of T. serrulatus, including chitinase, peptidyl-α-hydroxyglycine α-amidating lyase (PAL), peptidyl-glycine α-amidating monooxygenase A (PAM), and peptidylglycine α-hydroxylating monooxygenase (PHM). These findings shed light on the diverse array of bioactive molecules present in T. serrulatus venom.

### Metalloproteases

Regarding proteases, metalloproteases are the most commonly present in animal venoms [55] and need a cofactor to perform the proteolytic activity, such as bivalent ions [56,57]. Some studies have identified the presence of metalloproteases in the venom of T. serrulatus. The study by Fletcher et al. [58] characterized a new metalloproteinase called antarease, which cleaves vesicle-associated membrane protein 2 (VAMP2) close to the transmembrane domain. VAMP2 is a protein that, along with the synaptosome-associated protein of 25 kDa (SNAP25) and Syntaxin, is essential for the release of a variety of biologically active molecules via exocytosis [59]. Zornetta et al. [60] produced a recombinant antarease from T. serrulatus venom and observed that it caused paralysis of the neuromuscular junction of insects and mammals, and they also indicated that this enzyme could act in voltage-gated calcium channel, inactivating it. Venancio et al. [61] identified dynorphin-cleaving metalloproteinases that may be antarease-like molecules.

The action of metalloproteases may be related to the acute pancreatitis that occurs in scorpion stings [6], which has already been reported. Machado and Silveira-Filho [62] showed hemorrhagic pancreatitis caused by the T. serrulatus toxin in dogs, while Novaes et al. [63] observed acute pancreatitis in rats after the injection of T. serrulatus toxin. Gallagher, Sankaran, and Williams [64] demonstrated that the scorpion venom indirectly prompted the release of amylase by acting on nerve endings to release neurotransmitters.

Carmo et al. [55] identified ten proteases named metalloserrulases (TsMs), which showed similarities (from 46 to 95%) with the antarease sequence. These TsMs have a zinc-binding site and a conserved methionine, a common structure in the metzincin family, except for TsMs 10, which presents a great similarity with gluzincins and M13 metalloprotease families [55]. Metzincins are related to proteases A Disintegrin and Metalloprotease (ADAM) family, which in snakes are directly involved with the envenoming and blood clotting process [65]. Gluzincins are angiotensin-converting enzyme-like, and are involved in biological processes related to the conversion of angiotensin I into II [49,66]. Additionally, Carmo et al. [55] also reported that these proteases are involved in the maturation process of other toxins present in the venom, cleaving near arginine and lysine residues, which is also demonstrated by Martin-Eauclaire et al. [67] concerning the median lethal dose (LD_50_) of different venom toxins.

The number of putative components in the transcriptome of T. serrulatus representing metalloproteases is considerable (~30%) [49,54] and in the proteome as well (~20%) [8]. However, a significantly larger amount of venom is required to detect any proteolytic activity, compared to snake venoms, for example [55]. 

**Figure 4.**
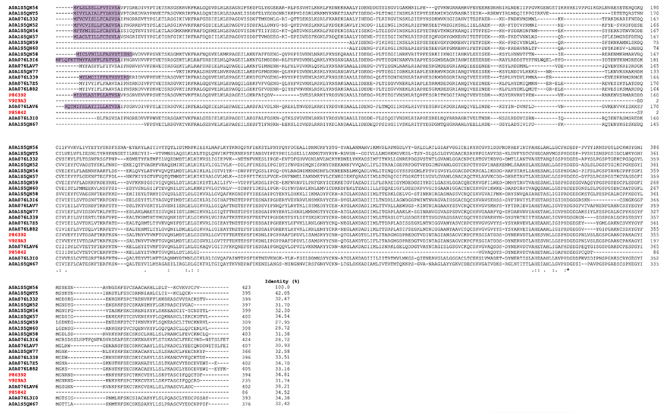
Multiple align among metalloproteases from Tityus serrulatus scorpion venom. Align among antarease (P86392), antarease-like (V9Z9A3), metalloprotease (P85842) metalloserrulase 2 (A0A076LAV6), 3 (A0A076L3I0), 4 (A0A076L332), 5 (A0A076L7Z5), 6 (A0A076L882), 7 (A0A076LAV7), 8 (A0A076L3I6), 9 (A0A076L339), 11 (A0A1S5QN60), 12 (A0A1S5QN59), 13 (A0A1S5QN77), 14 (A0A1S5QN54), 15 (A0A1S5QN58), 16 (A0A1S5QN57), 17 (A0A1S5QNT5), 18 (A0A1S5QN52), 19 (A0A1S5QN56) and 20 (A0A1S5QN67). Red ID represents data from UniProtKB/Swiss-Prot, while black ID represents data from UniProtKB/TrEMBL. The purple box represents the signal peptide. Amino acid residues are indicated in black. *: fully conserved residues; :: residues with very similar properties; .: residues with dissimilar properties [68].

Although metalloproteases share the same phylogenetic origin [49,55], Figure 4 illustrates a comparison among various metalloproteases, including antarease, antarease-like, metalloprotease, and metalloserrulases. While there are similarities between some of them, only one amino acid residue is common to all of these proteases, with few displaying any significant similarity. 

### Serine proteases

Although gangrene, hemolysis, and necrosis are infrequently documented in human envenomation cases caused by T. serrulatus, these manifestations can occur in animals, indicating the presence of proteolytic enzymes within the venom [69]. Almeida et al. [70] identified enzymes that provided gelatinolytic activities in vitro, which are potentially serine proteases, because they were inhibited by phenylmethylsulphonyl fluoride (PMSF), a serine protease inhibitor, and their optimal pH was eight, the same for serine proteases [71]. Amorim et al. [8] also detected serine protease activity using the Fraction I from T. serrulatus. It is important to emphasize that this component was only identified in venom transcriptomics and proteomics [49].

### Hyaluronidases

Hyaluronidases are enzymes able to degrade hyaluronic acid, the major component of the extracellular [72], and are involved in several physiological and pathological activities such as fertilization, wound healing, embryogenesis, angiogenesis, diffusion of toxins and drugs, metastasis, pneumonia, sepsis, bacteremia, meningitis, inflammation, allergy, and others [73]. Being present in many animal venoms [73] and widely identified in scorpions [61,74,75], their major role is the facilitation of venom spread in the victim's tissues [76]. 

Hyaluronidase was isolated from T. serrulatus venom by Pessini et al. [77], which can confirm the spreading effect performed by this enzyme. Furthermore, the presence of this component may be related to the lethality of the venom. Horta et al. [78] produced an anti-hyaluronidase antibody from T. serrulatus, which inhibited the enzyme's action both in vitro and in vivo, effectively reducing the venom's toxicity. The same antibody was used by Oliveira-Mendes et al. [79], who demonstrated that hyaluronidase not only played a crucial role in venom spreading but also inhibiting it, resulting in a delay in venom biodistribution from the bloodstream to target organs (e.g., lungs and liver), being this inhibitor a potential and a valuable first-aid agent for this type of envenoming.

**Figure 5.**
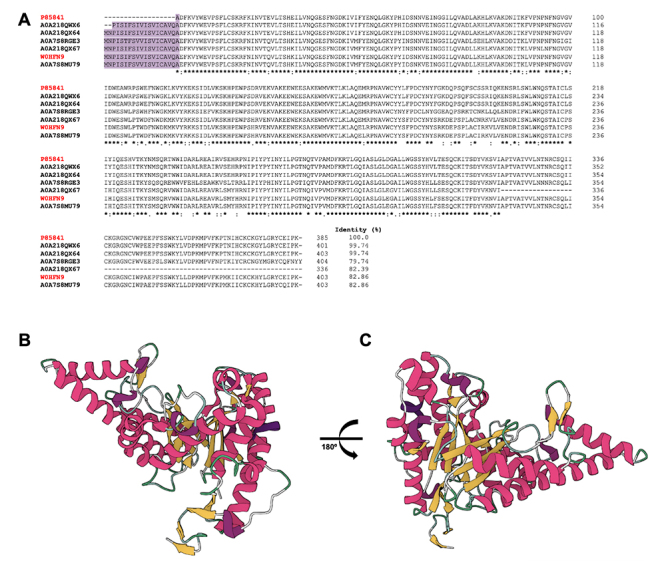
Structures of hyaluronidase from Tityus serrulatus scorpion venom. **(A)** Multiple align among hyaluronidases P85841, W0HFN9, A0A218QWX6, A0A218QX64, A0A7S8RGE3, A0A218QX67 and A0A7S8MU79. Red ID represents data from UniProtKB/Swiss-Prot, while black ID represents data from UniProtKB/TrEMBL. The purple box represents the signal peptide. Amino acid residues are indicated in black. *: fully conserved residues; :: residues with very similar properties; .: residues with dissimilar properties [68]. **(B)** Front and **(C)** back view of hyaluronidase-1 (P85841) structure, based on the amino acid sequence using Alphafold [80,81]. α-helix and β-sheet are represented in pink and yellow, respectively.

The structures of hyaluronidases are already deposited in the UniProtKB database [68], demonstrating that the six deposited sequences show high identity among them (> 79%) (Figure 5 A ). Additionally, the molecular model of hyaluronidases exhibits secondary structures, such as α-helix and β-sheets (Figure 5 B-C ), indicating the presence of different epitopes distributed throughout the molecule's structure, as demonstrated by Horta et al. [78]. In this study, the authors performed a systematic mapping of continuous epitopes which were recognized by anti-hyaluronidase serum with three antigenic regions common to both hyaluronidases TsHyal-1 and TsHyal-2 and could identify among these regions, the active site D^101^ and E^103^. Also, the three antigenic regions were mapped onto the 3D models of both hyaluronidases and were found to surround the active sites, which could indicate that the neutralization of Ts venom by anti-hyaluronidase serum was a result of the binding of serum antibodies to specific residues in the Ts hyaluronidase active site [78].

### Phospholipases

Phospholipases are enzymes that hydrolyze steric bonds of phospholipids, that could infer in the membrane function and structure [82]. They can be involved in phospholipid metabolism, signal transduction, or other cellular functions, or extracellular when they are present in mammalian pancreatic juice and animal venom and act as platelet aggregators in the blood or as catalysts in the release of arachidonic acid, triggering inflammatory reactions [83]. 

Although PLA_2_ activity was not detected in fraction I of T. serrulatus venom, Amorim et al. [8] detected phospholipases in the venom proteome. In addition, De Oliveira et al. [49] observed the presence of transcripts of PLA_2_, PLC, and PLD, without proteomic evidence.

### Cysteine-rich secretory protein

**Figure 6.**
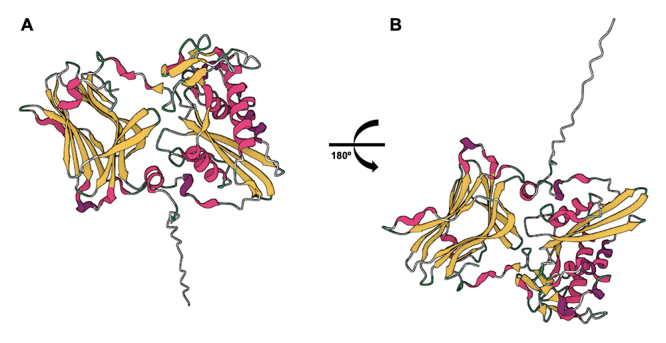
Structure prediction of CRISP from Tityus serrulatus scorpion venom. The structure was predicted through the amino acid sequence of CRISP (A0A218QX58) using Alphafold [80,81]. **(A)** Front and **(B)** back view. α-helix and β-sheet are represented in pink and yellow, respectively.

CRISP was also identified in T. serrulatus venom, and its role is still unclear [8]. However, CRISPs are widely distributed in animal venoms, such as snake venoms, being their role on ion channels also demonstrated [84], and in humans, they are involved with the immune system [85]. The molecular model of CRISP is demonstrated in Figure 6 A-B . Despite the presence of these compounds in proteomic and transcriptomic approaches of T. serrulatus venom [8,49], their activity was not detected in some articles [61,74,75], with a need for further study on these molecules and their presence in the venom.

### Others

Phosphodiesterases are enzymes that hydrolyze cyclic nucleotides and play a role in regulating intracellular levels of cyclic adenosine monophosphate (cAMP), cyclic guanosine monophosphate (cGMP) and, therefore, cell function [86,87]. Its presence in the venom was detected by proteome [8].

PAL, PAM, and PHM were found in the transcriptome analysis [52], and these enzymes are responsible for post-translational modifications of venom toxins, such as C-terminal amidation, which plays a fundamental role in enhancing their lethal effect [48,88,89]. 

Chitinases play a crucial role in the digestive process of T. serrulatus by being present in its intestinal system [90]. Consequently, identifying these enzymes in the transcriptome could be directly linked to the effective digestion of prey organisms [52].

Identifying antarease, metalloproteases, peptides rich in cysteine, and phospholipases highlights the venom's potential enzymatic activity and its role in disrupting various physiological processes. Moreover, the presence of hyaluronidase suggests a possible involvement in tissue degradation and facilitating venom spread, while CRISP proteins may contribute to modulating the victim's immune response. PDE identification is noteworthy as this enzyme can impact intracellular signaling pathways. Some described components are also involved with the enhancement of the lethality of toxins, as well as the prey’s digestion. These studies have significantly improved our understanding of the T. serrulatus venom composition overall by identifying and characterizing several important venom components. Further research building upon these findings can contribute to developing novel therapeutic interventions and enhancing our knowledge of the molecular mechanisms underlying envenomation.

## Conclusion

T. serrulatus envenoming is of great medical importance in Brazil, and it can be more severe and frequent in children and patients with comorbidities. Antivenom treatment is available in healthcare services and recommended for moderate and severe cases. Notably, T. serrulatus scorpion venom is an extraordinary source of proteins with different molecular weights and performing different roles. The high molecular weight components can play crucial roles during the T. serrulatus envenomation strategy and have evolved to subdue prey or defend against predators effectively; thus, some considerations can be inferred. Many of these components in T. serrulatus venom are enzymatic proteins and play various functions, such as facilitating the breakdown of tissues, interfering with physiological processes, disrupting the prey’s defense mechanisms, increasing the lethality of toxins, or helping the digestion of prey. Enzymes identified in T. serrulatus venom often have larger molecular weights due to their complex structures and functional domains, exhibiting complex tertiary structures, which could provide stability and protection against degradation.

Additionally, these components in T. serrulatus venom can participate in intricate interactions with target molecules in the prey or victim's body, may involve binding to specific receptors or interfering in signaling pathways, targeting different physiological systems or employing multiple mechanisms of action simultaneously, increasing their chances of subduing prey or defending themselves effectively, suggesting the enhancement of venom's potency. Although the high molecular weight components were identified in T. serrulatus venom, and some of them were isolated, further research into the venom's composition and function can provide deeper insights into the precise roles of these components and their impact on envenomation.

## Abbreviations

ADAM: A Disintegrin and Metalloprotease; ALT: alanine aminotransferase; AST: aspartate aminotransferase; cAMP: cyclic adenosine monophosphate; cGMP: cyclic guanosine monophosphate; CPK: creatine phosphokinase; CRISP: cysteine-rich secretory protein; LD_50_: median lethal dose; MLD: minimum lethal dose; NO: nitric oxide; PAL: peptidyl-α-hydroxyglycine α-amidating lyase; PAM: peptidyl-glycine α-amidating monooxygenase A; PDE: phosphodiesterase; PHM: peptidylglycine α-hydroxylating monooxygenase; PLA_2_: phospholipase A_2_; PLC: phospholipase C; PLD: phospholipase D; PMSF: phenylmethylsulphonyl fluoride; SNAP25: synaptosome-associated protein of 25 kDa; TsMs: metalloserrulases; VAMP2: vesicle associated membrane protein 2.

## Data Availability

Not applicable.
